# Mandibular Condyle Osteomyelitis Following Third Molar Extraction: A Case Report and Literature Review

**DOI:** 10.7759/cureus.98860

**Published:** 2025-12-09

**Authors:** Rafael Castro Nobre, Joana Silva, Andreia Ferreira, Rute Saleiro, André S Luís

**Affiliations:** 1 Maxillofacial Surgery Department, Centro Hospitalar Universitário de Santo António, Porto, PRT

**Keywords:** mandibular condyle, maxillofacial surgery, odontogenic infection, osteomyelitis, parvimonas micra, prevotella nigrescens, temporomandibular joint, tmj osteomyelitis

## Abstract

Osteomyelitis of the mandibular condyle is an exceptionally rare but clinically significant infection, usually due to odontogenic sources. We report a case of a 33-year-old healthy male patient who developed condylar osteomyelitis after the extraction of an impacted lower right third molar (tooth 48). Seven days after the extraction, the patient reported increasing pain and trismus, and an initial trial of a muscle relaxant provided no clinical benefit. Maxillofacial CT scan revealed a 6 cm abscess along the medial aspect of the right mandibular ramus. Dual oral antibiotics (amoxicillin/clavulanate and metronidazole) were started, but a control CT scan showed an unchanged collection, prompting incision and drainage with pus sampling. Microbiology isolated *Parvimonas micra* and *Prevotella nigrescens* (susceptible to amoxicillin/clavulanate, metronidazole, and meropenem; resistant to clindamycin). The patient received 12 days of intravenous antibiotics and physiotherapy, with marked clinical improvement. After discharge, he continued long-course oral antibiotics for four weeks. At the six-month follow-up, the patient was asymptomatic, with full pain-free temporomandibular joint (TMJ) mobility and mouth opening >45 mm. The final CT scan demonstrated restitution of normal cortical continuity and marked reduction of previous bone rarefaction, consistent with spontaneous bone regeneration of the mandibular condyle. To deepen our understanding, we conducted a PubMed literature review (2000-2024; English; condylar involvement; abstracts available), which yielded 54 records and 20 relevant articles. Odontogenic infection was the most frequent etiology, followed by tuberculous and otologic/medication-related osteonecrosis of the jaw (MRONJ)‑related causes. CT typically reveals an osteolytic, eroded condyle; management combines drainage/debridement and prolonged antibiotics (6-8 weeks), reserving condylectomy for refractory or necrotic cases. This case underlines the value of early imaging and culture‑guided therapy to preserve TMJ function and avoid ankylosis or deformity.

## Introduction

Osteomyelitis of the jaws remains a clinically significant but increasingly uncommon condition in the modern era. Its incidence has declined substantially not only due to widespread access to antibiotics but also because of improvements in nutrition, general health, oral hygiene practices, and earlier recognition and management of odontogenic infections. Nevertheless, mandibular osteomyelitis continues to occur more frequently than maxillary disease, largely because of the mandible’s comparatively poor vascularity and dense cortical structure, which favors bacterial persistence and chronicity. Within mandibular sites, involvement of the condylar process is exceptionally rare, representing only 1-3% of all reported mandibular osteomyelitis cases, according to published case series and reviews [1-20]. This scarcity is attributed to the condyle’s robust arterial supply - primarily from the maxillary artery and deep temporal branches - and to its relative distance from common odontogenic sources of infection, which typically originate in the dentoalveolar region.

Despite its rarity, osteomyelitis of the mandibular condyle poses a unique diagnostic and therapeutic challenge. Its early manifestations - preauricular pain, trismus, swelling, or malocclusion - overlap substantially with more common temporomandibular joint (TMJ) disorders, including capsulitis, synovitis, myofascial pain syndrome, and septic arthritis. Consequently, delayed diagnosis is common, and such delays may allow progression from an acute medullary infection to cortical destruction, periosteal reaction, sequestration, and ultimately, ankylosis. Timely recognition is therefore essential to avoid irreversible functional impairment.

The etiology of condylar osteomyelitis is diverse. Although odontogenic infection is the predominant cause reported in the literature, other infectious sources - including tuberculosis (TB) [2,3,5,8,11,15,18], medication-related osteonecrosis of the jaw (MRONJ) [6], otologic infections [16], suppurative parotitis [13], actinomycosis [10], and even ectopic third molars [4] - have been described. The increasing number of TB-related cases in recent decades emphasises the need for clinicians to consider atypical pathogens, especially in patients with chronic symptoms, immune compromise, or exposure risk. Polymicrobial anaerobic infections remain typical in pyogenic cases, with organisms such as *Parvimonas micra*, *Prevotella* spp., and *Actinomyces* spp. frequently implicated [4,9,10,17].

Radiologic assessment plays a central role in diagnosis. CT scanning remains the modality of choice for evaluating cortical erosion, lytic defects, and sequestra [1,4,7,9], while MRI provides superior assessment of marrow edema, soft-tissue extension, and synovial involvement. In early or ambiguous cases, bone scintigraphy has been reported as a sensitive adjunct for identifying active inflammatory sites [1]. In pediatric patients, longitudinal imaging is crucial, given the risk of condylar growth disturbance and hypoplasia [11,12,19].

Management strategies typically follow a stepwise escalation model: early drainage of abscesses, prolonged culture-guided antibiotic therapy, and surgical debridement or condylectomy when structural compromise or persistent necrosis is present [1,2,13,14,16,20]. However, recent reports describe full resolution with conservative therapy - including drainage, targeted antibiotics, and physiotherapy - without condylar resection [4,7,9,10]. Spontaneous condylar regeneration following sequestrectomy has also been documented, highlighting the inherent osteogenic potential of the periosteum [20].

We present a case of odontogenic osteomyelitis of the mandibular condyle caused by strict anaerobes, followed by a focused literature review (2000-2024), with the objective of highlighting the diagnostic challenges, therapeutic decision-making, and potential for complete functional and radiologic recovery with conservative management.

## Case presentation

**Figure 1:**
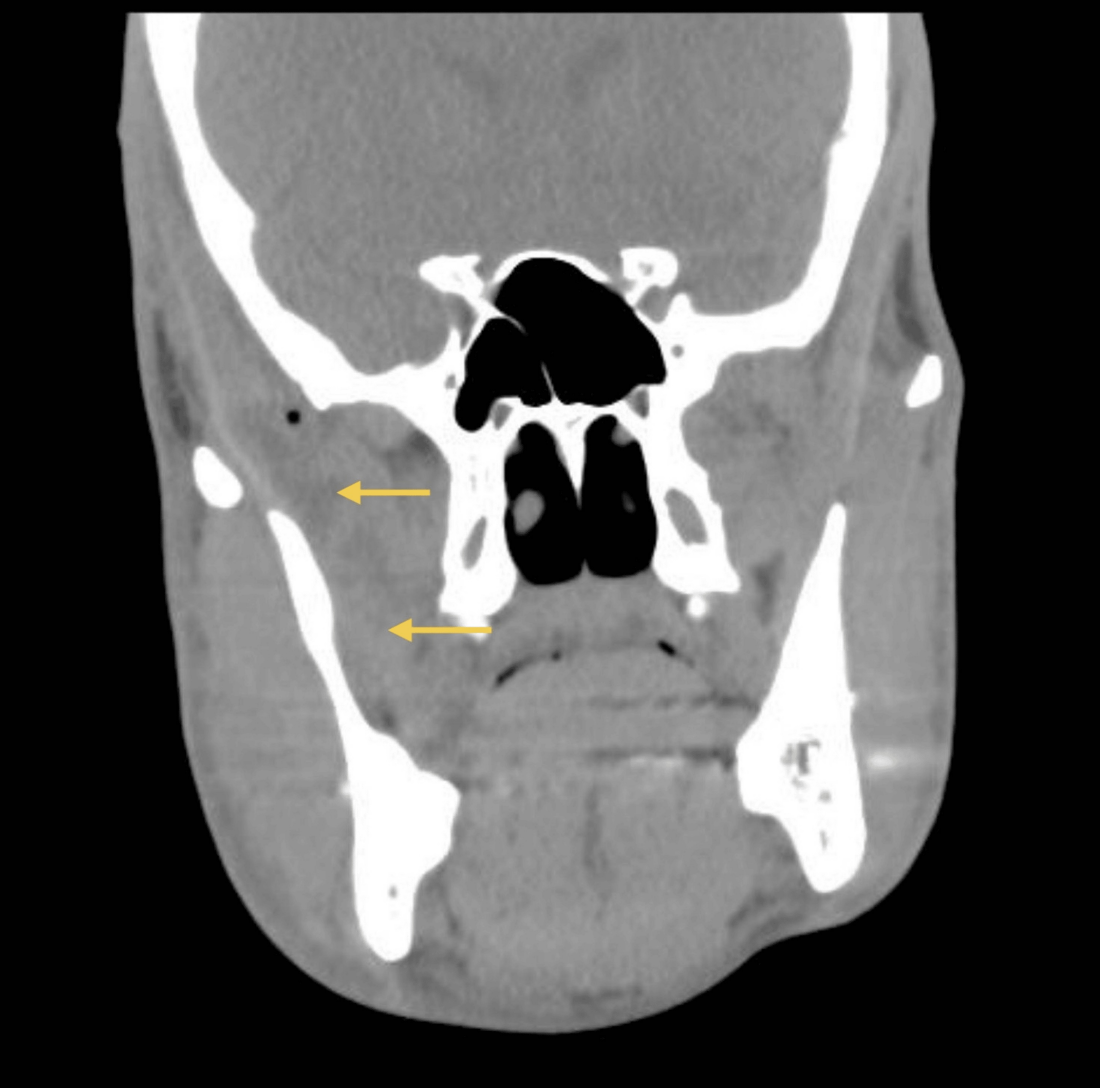
CT scan (coronal view) at emergency department Liquid collection abutting the medial surface of the right mandibular ramus, measuring 6 cm craniocaudally. The yellow arrows indicate the abscess collection.

**Figure 2:**
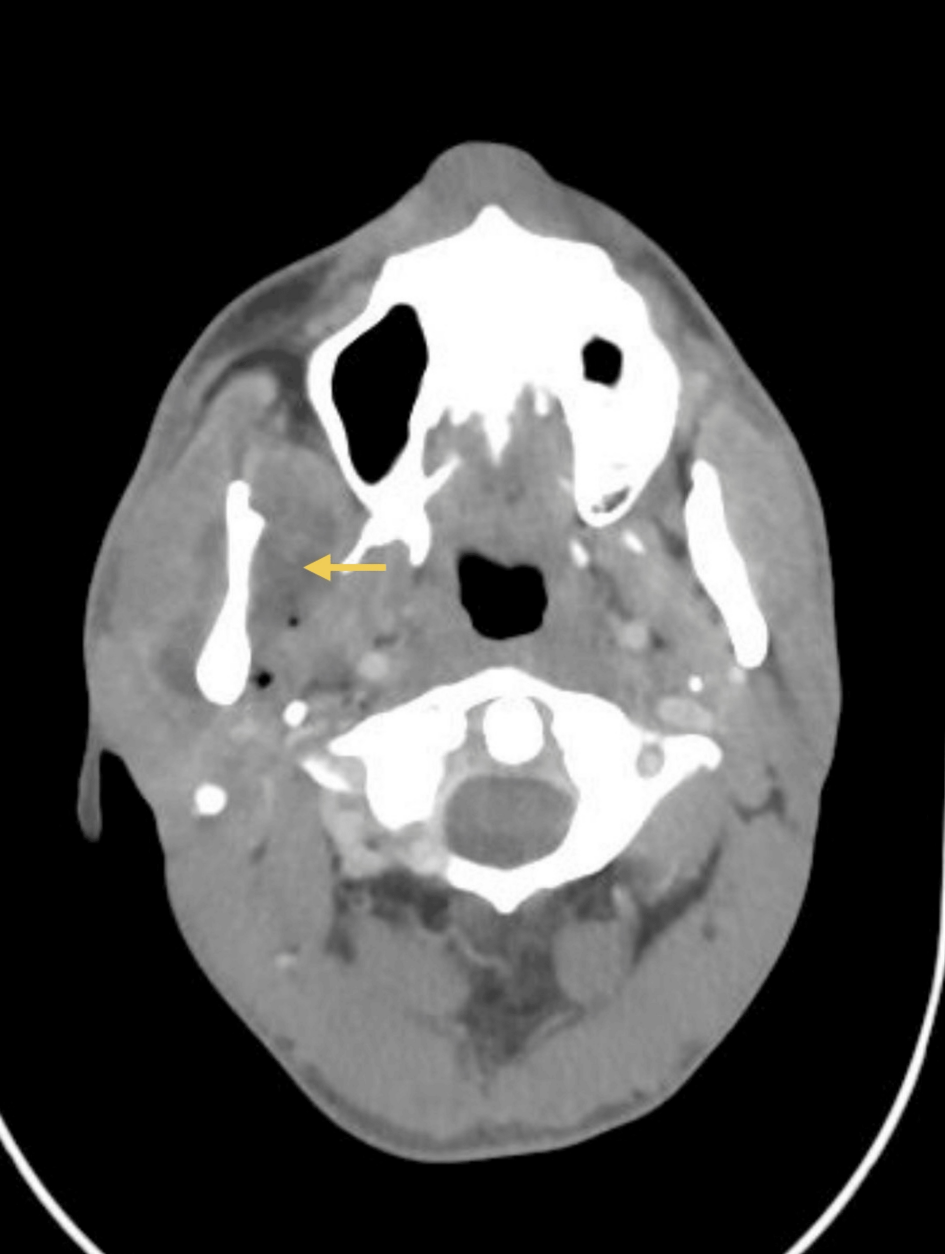
CT scan (axial view) at emergency department Liquid collection abutting the medial surface of the right mandibular ramus, measuring 6 cm craniocaudally. The yellow arrow indicates the abscess collection.

A 33-year-old healthy male patient, with no relevant medical history and no chronic medication, underwent extraction of an impacted lower right third molar (tooth 48) without intraoperative complications. Seven days later, he developed progressively worsening pain and limitation of mouth opening. His dentist prescribed a muscle relaxant, but symptoms continued to deteriorate. He went to the emergency department at Hospital Santo António, where he was observed by the maxillofacial surgery team. Clinical examination revealed trismus, mild right hemifacial edema, and tenderness at the mandibular angle. A CT scan (Figures 1-2) confirmed a liquid collection abutting the medial surface of the right mandibular ramus, measuring 6 cm craniocaudally. Given the patient’s age and overall good health, dual oral therapy (amoxicillin/clavulanate 875/125 mg 12/12h + metronidazole 500 mg 12/12h) was initiated with a planned reassessment.

**Figure 3:**
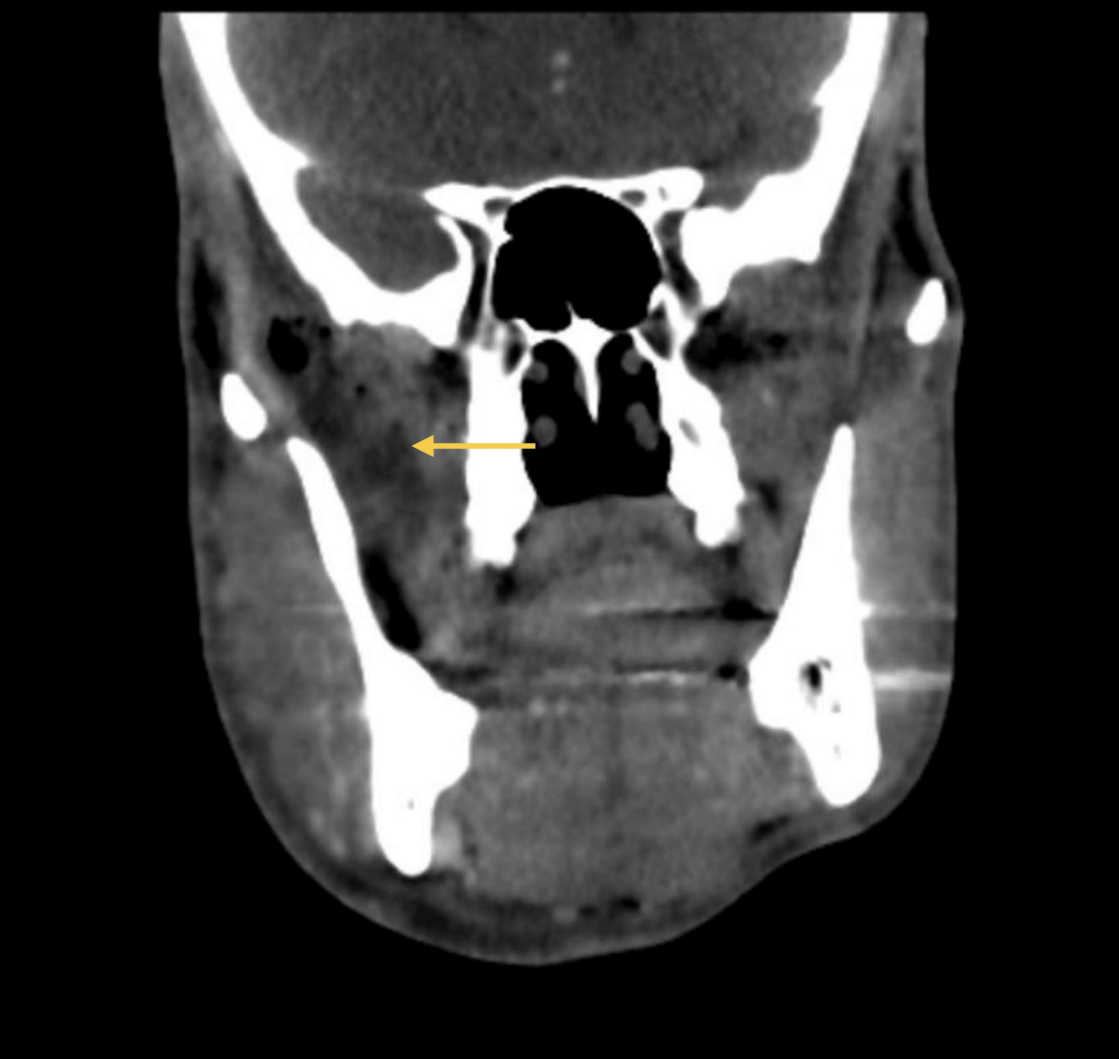
CT scan (coronal view) at admission Persistent abscess encasing the ramus and ipsilateral pterygoid muscles. The yellow arrow indicates the abscess collection.

**Figure 4:**
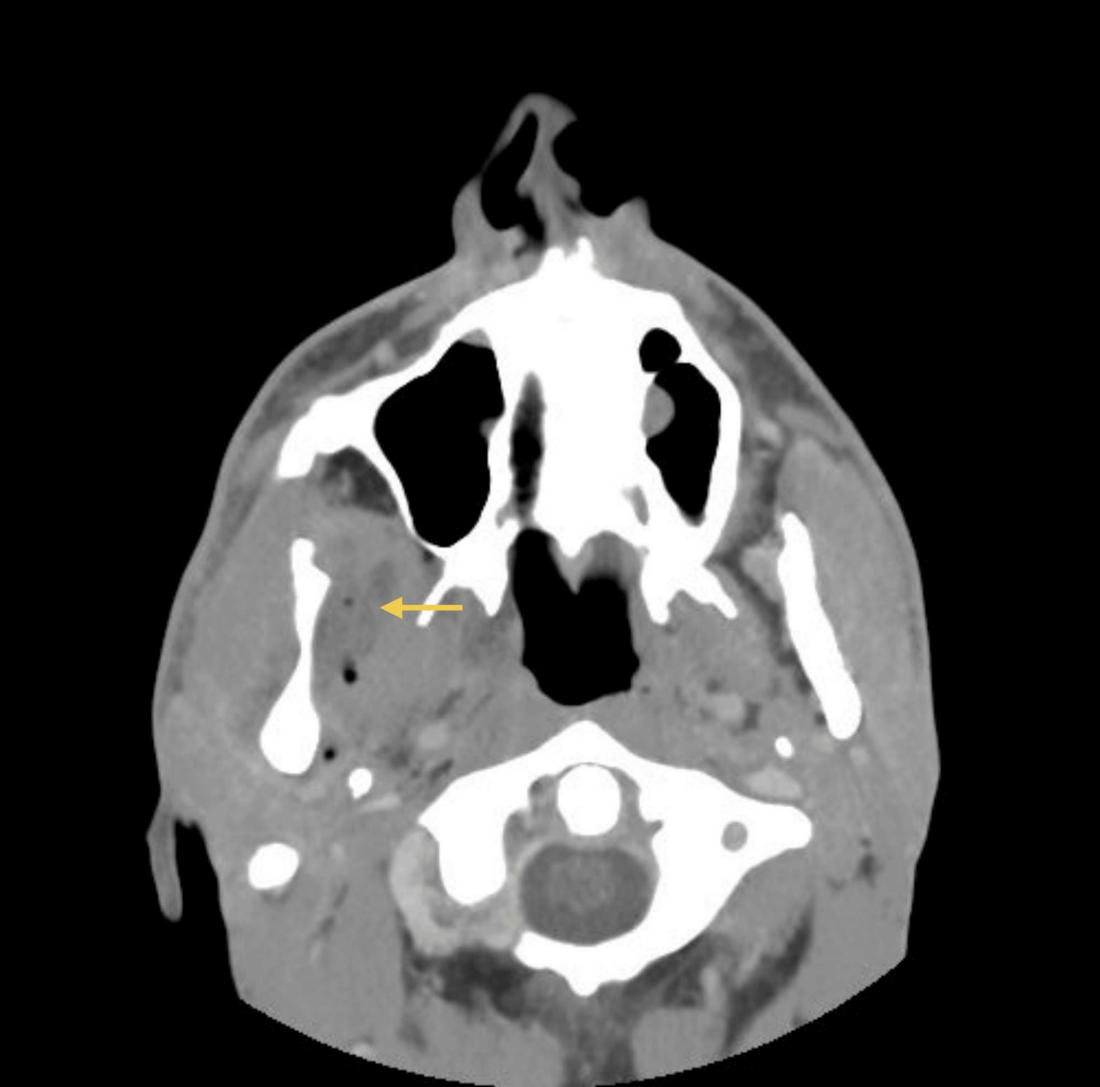
CT scan (axial view) at admission Persistent abscess encasing the ramus and ipsilateral pterygoid muscles. The yellow arrow indicates the abscess collection.

As symptoms worsened, a control CT scan (Figures 3-4) was taken, which showed a persistent abscess encasing the ramus and ipsilateral pterygoid muscles. Incision and drainage were performed under local anesthesia via a trans-oral approach to ensure safe access to the medial ramus. Care was taken to avoid injury to adjacent neurovascular structures, and purulent material was collected using sterile aspiration to prevent contamination and ensure optimal microbiological yield. Cultures isolated *Parvimonas micra* and *Prevotella nigrescens* (susceptible to amoxicillin/clavulanate, metronidazole, and meropenem; resistant to clindamycin). The patient was admitted for 12 days of intravenous amoxicillin/clavulanate 1200 mg 12/12h and metronidazole 500 mg 8/8h, along with a three-day course of dexamethasone and physiotherapy exercises. Reassessment performed 48 hours after admission showed clinical improvement, and therefore, the drain was removed.

**Figure 5:**
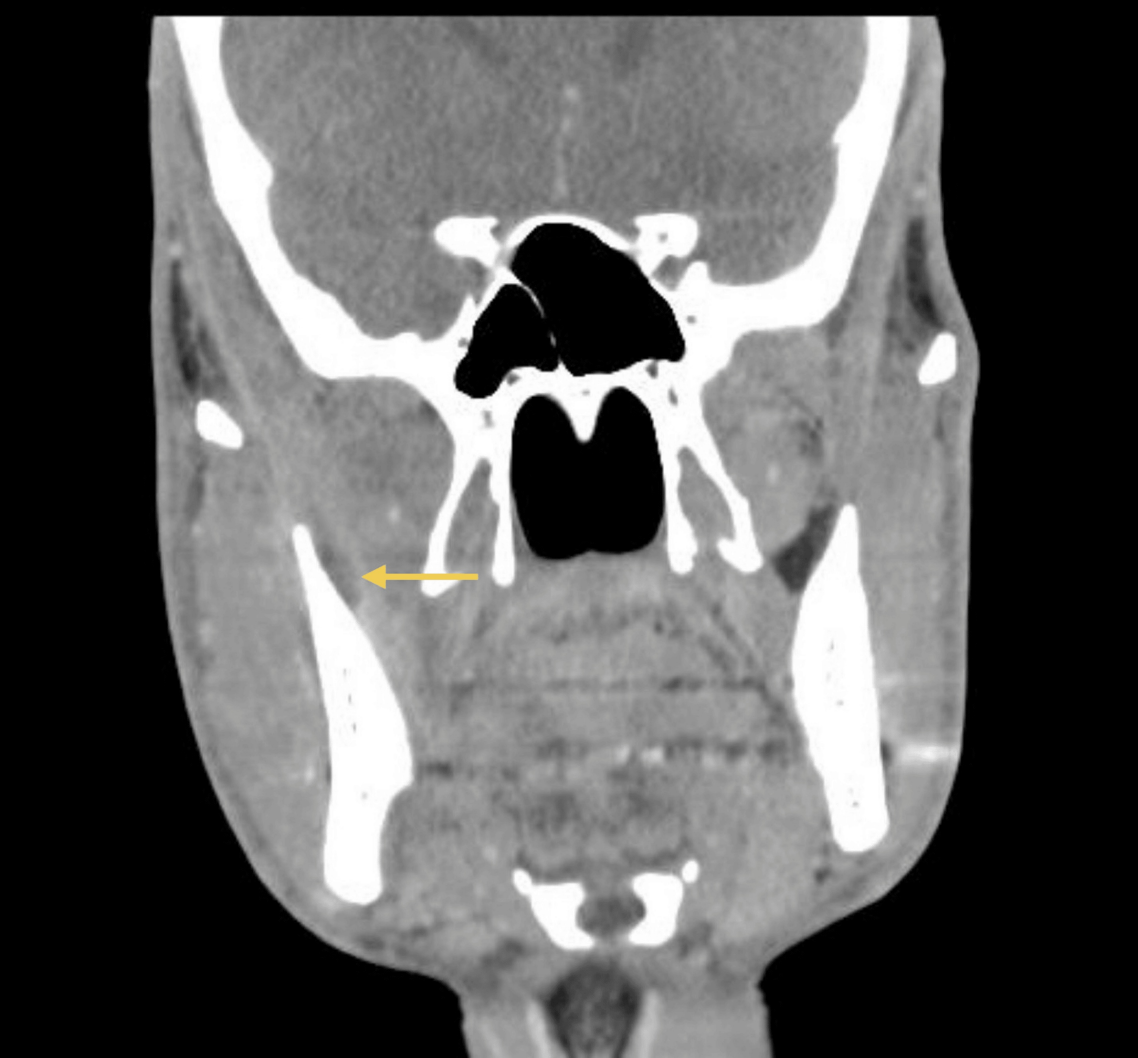
CT scan (coronal view) nine days after drainage Heterogeneity of the right mandibular ramus and condyle with irregular cortical surfaces compatible with osteomyelitis, plus a 10 mm anterior condylar collection. The yellow arrow indicates the abscess collection.

**Figure 6:**
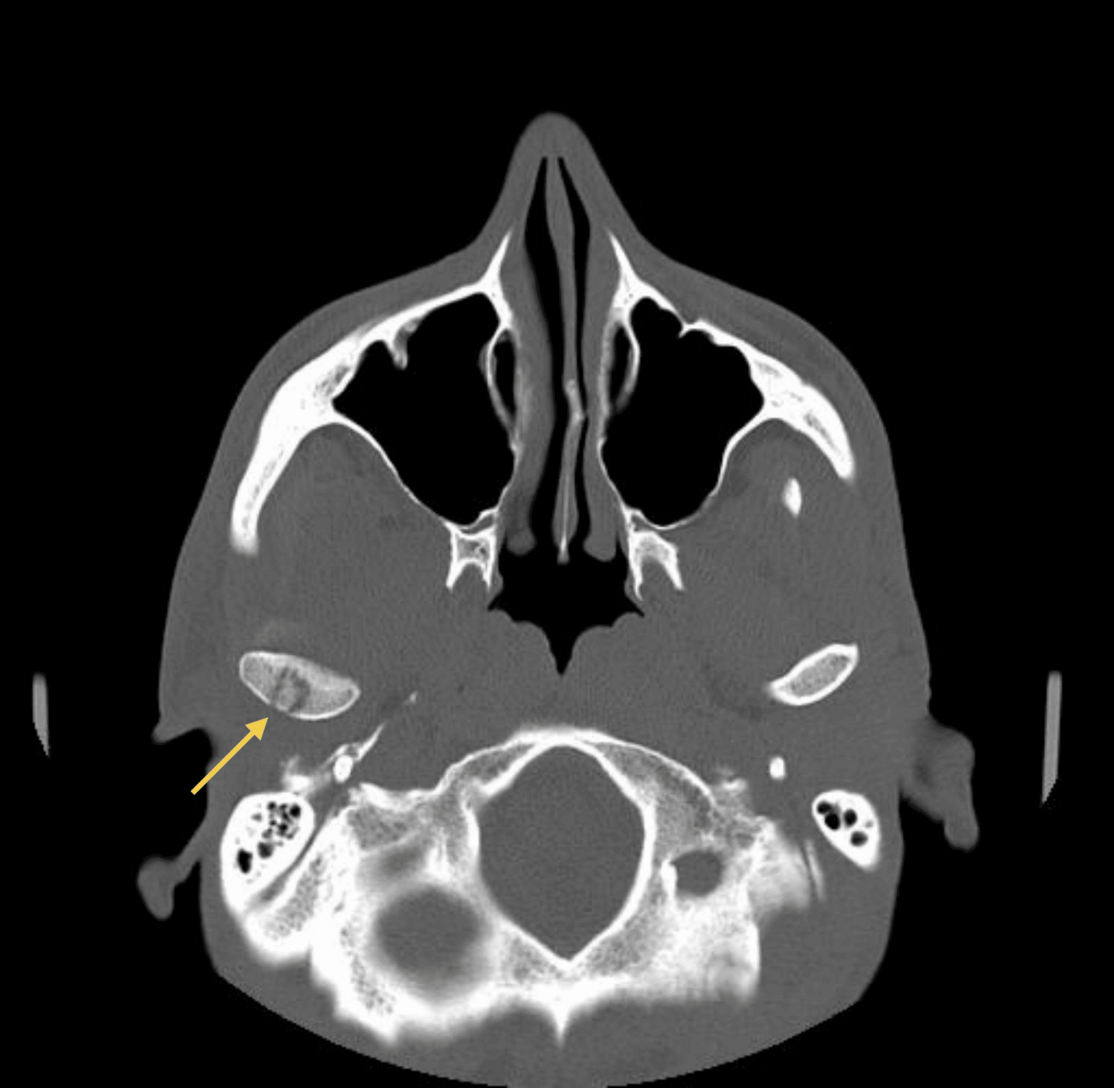
CT scan (axial view) nine days after drainage Heterogeneity of the right mandibular ramus and condyle with irregular cortical surfaces compatible with osteomyelitis, plus a 10 mm anterior condylar collection. The yellow arrow indicates the bone rarefaction of the mandibular condyle, consistent with osteomyelitis.

Nine days after drainage, a CT scan (Figures 5-6) displayed heterogeneity of the right mandibular ramus and condyle with irregular cortical surfaces compatible with osteomyelitis, plus a 10 mm anterior condylar collection.

**Figure 7:**
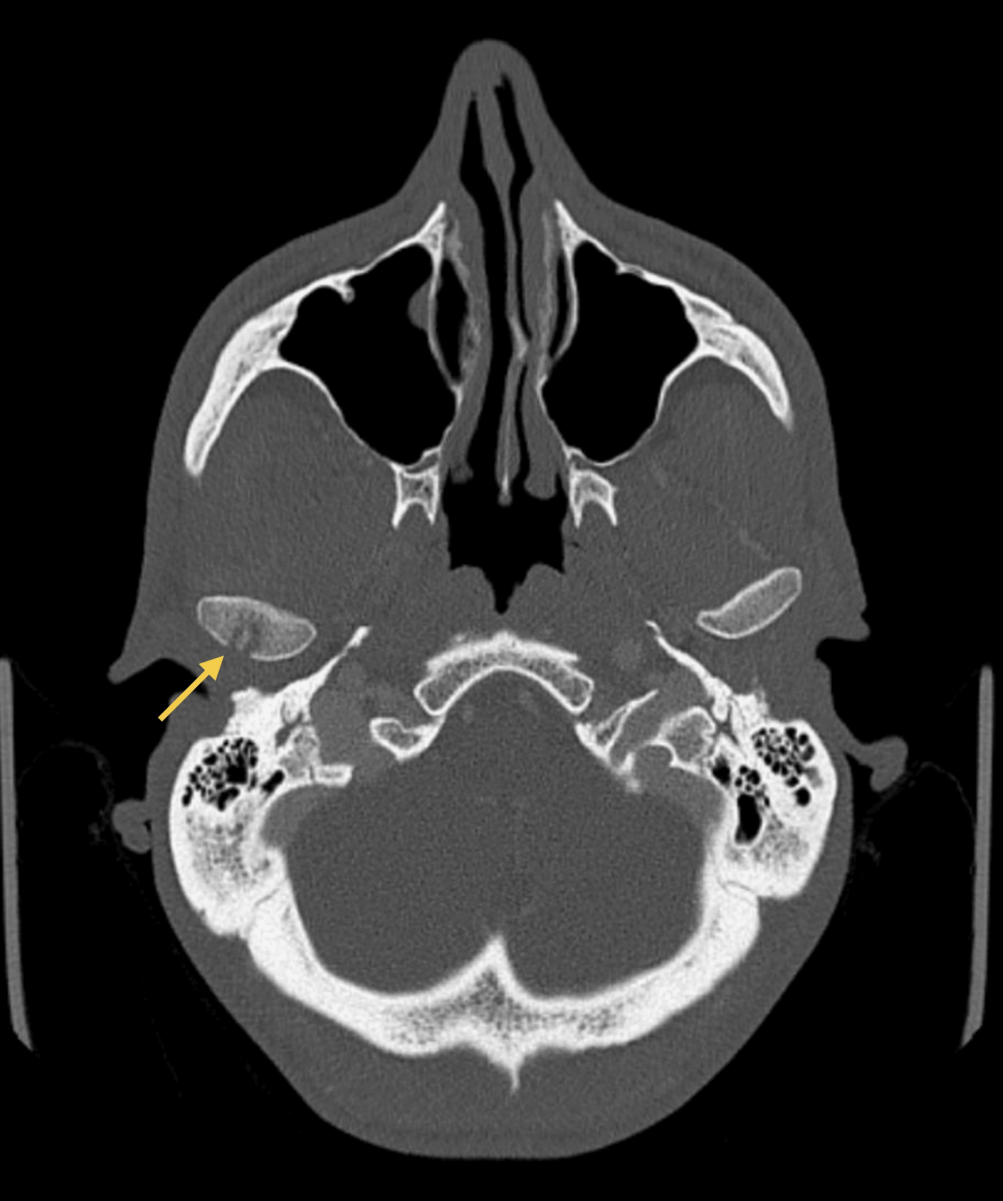
CT scan (axial view) at discharge Central osteolysis of the condylar head/neck with greater posterior cortical interruption and minor anterior cortical defects; no new collections. The yellow arrow indicates the bone rarefaction of the mandibular condyle, consistent with osteomyelitis.

A CT scan at discharge (Figure 7) demonstrated central osteolysis of the condylar head/neck with greater posterior cortical interruption and minor anterior cortical defects without any new collections. Long‑course oral antibiotics (amoxicillin/clavulanate 875/125 mg 12/12h + metronidazole 500 mg 12/12h) were continued for four weeks at home.

**Figure 8:**
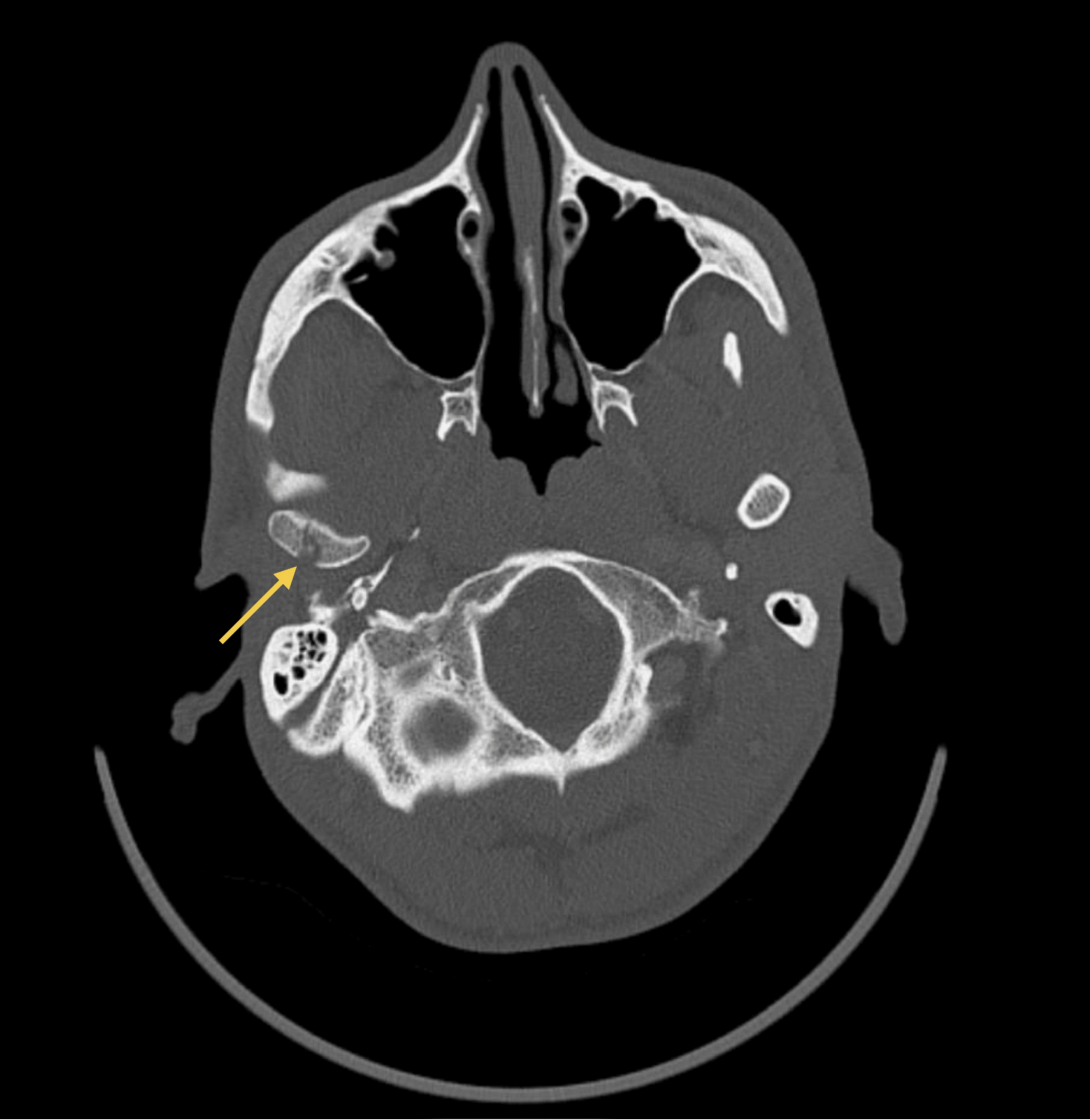
CT scan (axial view) four weeks after discharge Resolution of the prior abscesses and stable rarefaction of the condyle and ramus consistent with healing osteomyelitis. The yellow arrow indicates the bone rarefaction of the mandibular condyle, consistent with osteomyelitis.

At four weeks, a follow-up CT scan (Figure 8) showed resolution of the prior abscesses and stable rarefaction of the condyle and ramus consistent with healing osteomyelitis. Clinically, the patient improved steadily, achieving a pain‑free TMJ and maximal opening of 42 mm.

**Figure 9:**
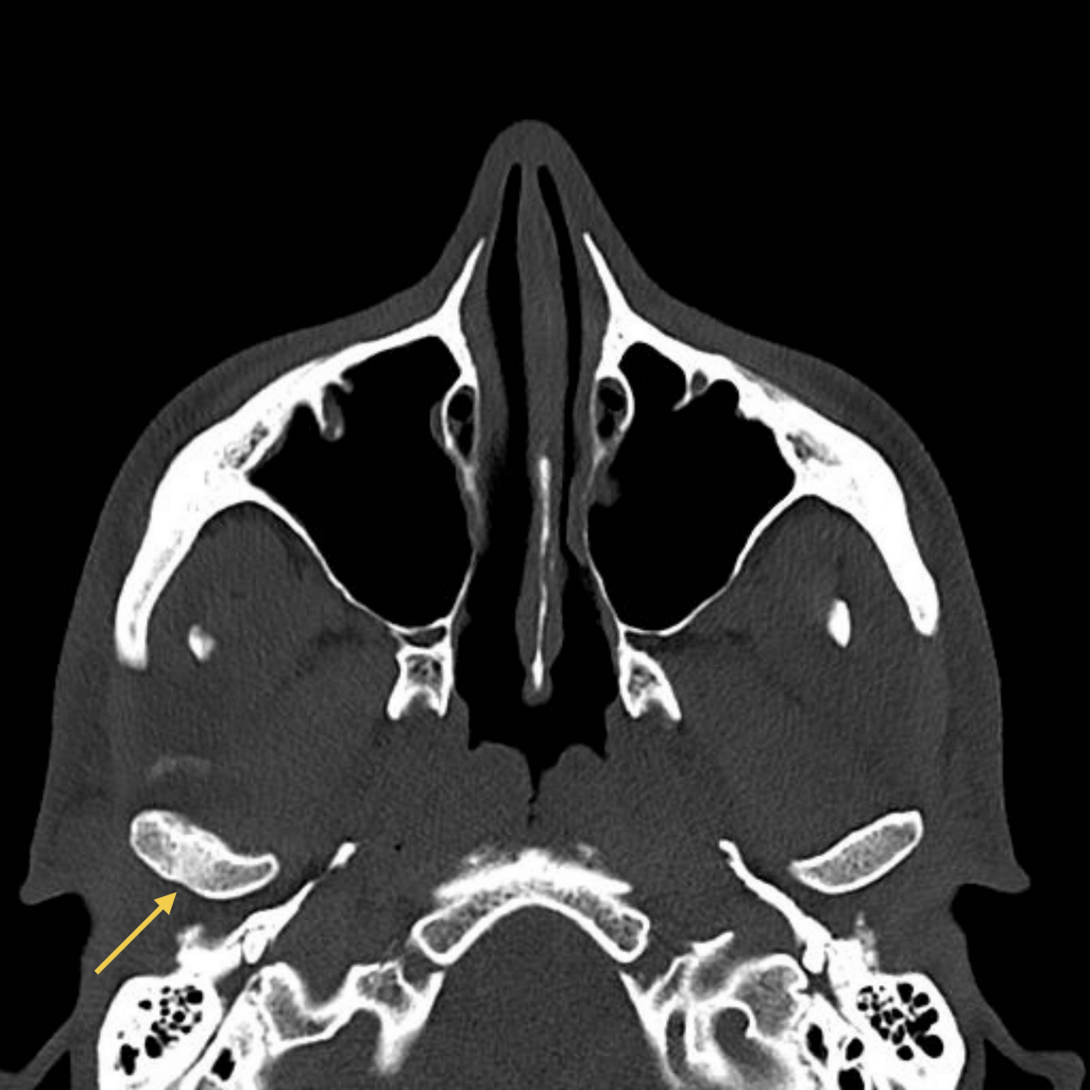
CT scan (axial view) at the six-month follow-up Bone regeneration of the condylar head, with reestablishment of cortical integrity and absence of rarefaction or periarticular thickening. The yellow arrow indicates the mandibular condyle showing bone regeneration.

At the six-month follow-up, the patient reported complete resolution of symptoms, with no pain, trismus, or TMJ discomfort and maximal mouth opening >45 mm. The final CT scan (Figure 9) showed clear evidence of condylar bone regeneration, with restored cortical integrity and absence of rarefaction or periarticular thickening. The patient was discharged from further follow-up at that time, after a total surveillance period of six months.

## Discussion

Condylar osteomyelitis is rare, likely due to its anatomical distance from common odontogenic sources and to the rich vascular supply of the temporomandibular region. The mandibular condyle receives arterial irrigation from multiple branches, including the deep auricular and anterior tympanic branches of the maxillary artery, the superficial temporal artery, the transverse facial artery, and periarticular capsular vessels, forming a dense anastomotic network. This extensive collateral circulation is thought to confer protection against infection compared to the more poorly vascularized mandibular body, consistent with the predominance of odontogenic osteomyelitis in non-condylar mandibular sites and the rarity of condylar involvement reported across published series [1,4,7,9].

Across recent reports, odontogenic infection remains the predominant etiology, while tuberculous involvement, MRONJ, and otologic sources - including post-mastoidectomy spread - are important alternative differentials [1-10].

CT scanning is essential for delineating osteolysis, cortical erosion, and sequestration, while MRI provides greater sensitivity for detecting bone marrow edema, soft-tissue involvement, and synovitis. Additionally, bone scintigraphy may demonstrate early inflammatory activity prior to overt radiographic changes.

Therapeutic strategy follows a step-up approach supported by multiple case reports and series [1,5,11-19]: (i) drainage of abscesses and culture-guided antibiotic therapy for 6-8 weeks in pyogenic osteomyelitis; (ii) debridement or sequestrectomy when necrotic bone is present; (iii) partial or total condylectomy reserved for refractory cases, extensive necrosis, or ankylosis risk.

The current case stands out not only due to the rare anatomic involvement but also for its spontaneous condylar bone regeneration following conservative management.

The rich vascular network supplied by the maxillary artery and pterygoid plexus usually protects the condyle from infection. However, in this case, the infection originated from an odontogenic focus (post-extraction site of tooth 48) that extended medially to the ramus and ascended to the TMJ through the fascial spaces. The pathogens identified - *Parvimonas micra* and *Prevotella nigrescens *- are strict anaerobes commonly found in the oral cavity, particularly associated with endodontic and periodontal infections. *Parvimonas micra* is a Gram-positive anaerobic coccus, implicated in chronic oral infections and occasionally in deep-space infections, septic arthritis, and vertebral osteomyelitis. Its virulence is linked to its capacity to form biofilms and synergize with Gram-negative anaerobes. *Prevotella nigrescens *is a Gram-negative anaerobic rod producing lipopolysaccharides and proteolytic enzymes that degrade connective tissue and bone. Together, these bacteria create a synergistic polymicrobial environment, promoting osteolytic destruction and chronic inflammation.

Current evidence supports a stepwise, conservative-first approach to condylar osteomyelitis: prompt drainage of abscesses to reduce bacterial load and intraosseous pressure [4,7,9,10], targeted antibiotic therapy guided by culture results for at least 6-8 weeks in pyogenic infection [1,5,11,17], physiotherapy to restore TMJ mobility and prevent ankylosis [9,10], and surgical intervention (partial or total condylectomy) reserved for refractory disease, extensive necrosis, or ankylosis risk [1,2,13,14,16,20].

In this case, the combination of timely drainage, culture-directed dual antibiotic therapy, and functional rehabilitation achieved complete clinical and radiological remission without the need for condylar resection.

Most published cases report favorable outcomes when infection is recognized early and managed through a multidisciplinary approach, as evidenced across numerous reports of odontogenic, tuberculous, otologic, and actinomycotic condylar osteomyelitis [1-5,7-10,13-20]. Prognosis depends primarily on the timing of diagnosis, the extent of osseous necrosis, and the antimicrobial resistance profile of the pathogens involved. The current case exemplifies that anaerobic osteomyelitis of the condyle can fully resolve under conservative therapy when the structural integrity of the periosteum and joint is maintained [10,20].

Literature review

A literature search was conducted on PubMed covering the last 25 years (2000-2024; English) using “osteomyelitis of the mandibular condyle” OR “osteomyelitis temporomandibular”. Inclusion criteria were: mandibular condyle osteomyelitis; abstract available. Exclusion criteria were: non‑condylar mandibular osteomyelitis; non‑mandibular bones; no abstract. A total of 54 records were analyzed, resulting in a final sample of 20 articles (n = 20) that met all the preestablished criteria (Table 1).

**Table 1:** Literature review Summary of 20 articles (2000-2024), presenting etiology, imaging, primary treatment, and outcomes. ATT, anti-tubercular therapy; NSAIDs, non-steroidal anti-inflammatory drugs; TMJ, temporomandibular joint; MRONJ, medication-related osteonecrosis of the jaw; ZN, Ziehl-Neelsen; NR, not reported; CSO, chronic suppurative osteomyelitis; OM, osteomyelitis; TJR, total joint replacement (i.e., complete replacement of the TMJ)

Author (Year)	Main Etiology	Imaging Summary	Primary Treatment	Outcome/Follow-up
Raghani et al. (2023) [3]	Tuberculosis (parotid mimic)	Preauricular mass; atypical imaging	Surgery + ATT (details NR)	Not reported
Chaudhary et al. (2023) [11]	Tuberculosis (neonate)	Preauricular lesion; erosion; ZN positive	Drainage + ATT; later distraction	Tuberculosis cured; condylar hypoplasia treated
Gupta et al. (2022) [2]	Tuberculosis	Suppurative condylar osteomyelitis	Sequestrectomy + high condylectomy + ATT	Resolution with regeneration
Iwai et al. (2021) [10]	Actinomycosis + proliferative periostitis	Condylar osteolysis + reactive bone	Third molar removal + coronoidectomy + ampicillin (7.5 months)	No recurrence (7 years), regeneration
Kato et al. (2020) [6]	MRONJ spread to condyle	Mandibular body OM → condylar destruction	Antimicrobials; MRONJ management	Not reported
Towdur et al. (2018) [18]	Primary tuberculosis of TMJ	Mimicked arthritis/OM	ATT ± surgery (NR)	Not reported
Chattopadhyay et al. (2017) [1]	Unknown (2 cases)	Osteolytic/eroded condyle; scintigraphy useful	Condylectomy + antibiotics	Resolution
Vorrasi and Zinberg (2017) [13]	Suppurative parotitis	Condylar + parotid involvement	Resection + parotid drainage + IV antibiotics; TJR	No recurrence (24 months)
Seok et al. (2015) [12]	Proliferative periostitis	Mimicked osteogenic sarcoma	Decortication + anti-inflammatories (NR)	Not reported
Kumar et al. (2015) [5]	Tuberculosis	Radiolucency/condylar erosion	ATT (± surgery)	Clinical improvement
Kim (2015) [16]	Post-mastoidectomy (otologic)	TMJ/condyle osteitis	Resection + antibiotics (NR)	Not reported
Berglund et al. (2015) [19]	Primary chronic OM (children)	Some cases with collum/condyle	NSAIDs + decortication	Partial recurrence
Koul et al. (2014) [15]	TB mandibular OM	Mandibular destruction; retromandibular mass	ATT (9 months)	Marked reduction
Wang et al. (2014) [9]	Pericoronitis (third molar)	Condylar osteomyelitis	Extraction + drainage + two-week antibiotics	Full recovery
Lee et al. (2013) [14]	Chronic OM with intracranial spread	Condylar displacement; brain abscess	Partial mandibulectomy/condylectomy + meropenem (6 weeks)	Full recovery
Lambade et al. (2013) [4]	Ectopic third molar	Extraoral sinus; condylar osteomyelitis	Removal of ectopic tooth + antibiotics	Good outcome
Sheikh et al. (2012) [8]	Tuberculosis	Atypical imaging; diagnostic dilemma	ATT	Not reported
Pourdanesh et al. (2012) [20]	Chronic OM with condylar sequestrum	Sequestrum = condylar process	Sequestrectomy	Spontaneous regeneration (12 months)
Zemann et al. (2011) [7]	Primary (no focus)	Near-complete condylar destruction	Prolonged antibiotics (no surgery)	Stable at 4 years
Rajkumar et al. (2010) [17]	Recurrent CSO with fracture	Multiple mandibular lesions; pathological fracture	Sequestrectomy + resection of coronoid/condyle + antibiotics	Improved mouth opening

The analysis of the 20 published cases of mandibular condyle osteomyelitis (2000-2024) confirms that this entity remains exceptionally rare and heterogeneous in etiology. The odontogenic origin (following dental extractions, pericoronitis, or, more rarely, ectopic third molars) was the most prevalent cause, representing over half of all documented cases [1,4,9,17,20]. Tuberculous infection consistently appears as the second most frequent etiology, reported in multiple series and case reports [2,3,5,8,11,15,16,18], while other causes include bisphosphonate-related osteonecrosis (MRONJ) [6], actinomycosis [10], postoperative otologic spread [16], and suppurative parotitis [13]. This distribution reflects the need for a dual diagnostic approach: in low TB prevalence regions, an odontogenic focus should be considered first, whereas in patients with atypical symptoms or poor response to antibiotics, tuberculous osteomyelitis must be excluded.

Radiological findings are remarkably consistent across reports. CT scan typically demonstrates osteolysis, irregular cortical erosion, and occasionally extension to the mandibular ramus or glenoid fossa [1,4,7,9]. MRI adds sensitivity for detecting soft-tissue extension, synovitis, and intra-articular effusion, assisting in differentiating osteomyelitis from septic arthritis of the TMJ. In early or equivocal cases, bone scintigraphy has been reported as a sensitive adjunct for identifying active inflammatory sites [1]. In pediatric cases, longitudinal imaging is crucial given the risk of condylar growth disturbance and hypoplasia [11,12,19].

From a microbiological perspective, non-tuberculous osteomyelitis is predominantly polymicrobial and anaerobic, involving *Parvimonas micra*, *Prevotella* spp., and occasionally *Actinomyces *spp. [4,9,10,17]. One exceptional case involved *Staphylococcus aureus* in association with suppurative parotitis [14]. Several reports highlight clindamycin resistance, reinforcing the advantage of empiric combinations, such as amoxicillin/clavulanate plus metronidazole, later tailored to culture results [6,9,17]. In tuberculous forms, diagnosis relies on histopathology and polymerase chain reaction (PCR), with standard multidrug anti-tubercular therapy (ATT) leading to successful infection control and even condylar bone regeneration [2,5,11,15].

Treatment strategies converge toward a stepwise approach: (i) drainage of abscesses and sequestrectomy/debridement when necrotic bone is present; (ii) prolonged antibiotic therapy, typically 6-8 weeks for pyogenic infections and 6-9 months for tuberculous cases; (iii) surgical resection (partial or high condylectomy) reserved for refractory disease, extensive necrosis, or functional ankylosis [1,2,13,14,16,20].

Although several authors advocate early condylectomy for extensive lesions, recent reports describe complete recovery with conservative treatment, including drainage, targeted antibiotics, and physiotherapy, without condylar resection [4,7,9,10]. Moreover, spontaneous condylar regeneration after sequestrectomy has been documented [20], underscoring the osteogenic potential of the periosteum and supporting bone-preserving management, especially in young patients.

Outcomes across studies are favorable when surgical control of infection is combined with appropriate antibiotics and functional rehabilitation. The most frequent complications are: TMJ ankylosis, mandibular deformity, and condylar hypoplasia in children [11,19]. Rare but severe complications include intracranial extension and brain abscess formation [14]. Recurrences are typically associated with incomplete drainage, unrecognized necrosis, or insufficient antibiotic treatment duration [17].

Compared to published evidence, the present case aligns closely with best practice principles: early imaging, prompt surgical drainage, culture-directed antibiotic therapy, and TMJ physiotherapy, which resulted in full recovery without condylectomy. These results reflect contemporary trends that emphasize the multidisciplinary management of mandibular condyle osteomyelitis, guided by microbiology and with preservation of function.

## Conclusions

Mandibular condyle osteomyelitis, although rare, should be suspected in cases of persistent preauricular pain or trismus following third molar surgery. Early CT/MRI and microbiological cultures enable targeted therapy. Amoxicillin/clavulanate plus metronidazole provide broad and effective coverage for aerobic and anaerobic specimens when supported by susceptibility testing, whereas clindamycin monotherapy may fail in resistant strains. Conservative management, including abscess drainage, prolonged antibiotic therapy, and physiotherapy, can preserve the condyle, while condylectomy should be reserved for refractory disease, extensive necrosis, or cases with a high risk of ankylosis.
